# Supplementary material for: Sexual Dimorphism in Third Molar Agenesis in Humans with and without Agenesis of Other Teeth
Source: Biology (Basel). 2022 Nov 28;11(12):1725. doi: 10.3390/biology11121725 (PMC9774884; doi:10.3390/biology11121725)
Supplement: Supplementary file 1 [file biology-11-01725-s001.zip › biology-1957546-supplementary.pdf]

Article

Sexual Dimorphism in Third Molar Agenesis in Humans with and without Agenesis of Other Teeth

Ragda Alamoudi, Mohammed Ghamri, Ilias Mistakidis and Nikolaos Gkantidis

Supplementary tables

Supplementary Table S1. Number of missing third molars in male and female individuals with and without agenesis of teeth other than third molars.

**Third molar agenesis with agenesis of other teeth**				**Third molar agenesis without agenesis of other teeth**				**P-Value**		
Gender	Incidence	Average per individual	Total number of missing third molars	Gender	Incidence	Average per individual	Total number of missing third molars	Incidence	Average per individual	Total number of missing third molars
Males	65/133 (48.9%)	1.29 ± 1.59	172/532 (32.3%)	Males	25/133 (18.9%)	0.45 ± 1.10	60/532 (11.3%)	<0.001[1]	<0.001[2]	<0.001[1]
Females	89/170 (52.4%)	1.45 ± 1.60	246/680 (36.2%)	Females	37/170 (21.8%)	0.49 ± 1.07	84/680 (12.4%)	<0.001[1]	<0.001[2]	<0.001[1]
P-Value	0.548[1]	0.451[2]	0.328[1]	P-Value	0.568[1]	0.548[2]	0.592[1]	-	-	-

[1]Chi-square test, [2]Mann-Whitney test (adjusted level of significance $p < 0.01$)

Supplementary Table S2. Prevalence of tooth agenesis of each third molar in males and females with and without agenesis of teeth other than third molars.

	Tooth type	**With other teeth agenesis**			**Without other teeth agenesis**		
		Total missing teeth	Average per patient	Prevalence	Total missing teeth	Average per patient	Prevalence
**Female**	18	58/170	0.00 ± 0.50	34.11%	23/170	0.10 ± 0.30	13.53%
	28	60/170	0.00 ± 0.50	35.29%	18/170	0.11 ± 0.31	10.59%
	38	62/170	0.00 ± 0.50	36.47%	18/170	0.10 ± 0.30	10.59%
	48	66/170	0.00 ± 0.50	38.82%	25/170	0.15 ± 0.36	14.70%
	**P-value[2]**	0.827[1]	-	-	0.559[1]	-	-
**Male**	18	43/133	0.00 ± 0.50	32.33%	14/133	0.10 ± 0.30	10.53%
	28	45/133	0.00 ± 0.50	33.83%	14/133	0.11 ± 0.31	10.53%
	38	46/133	0.00 ± 0.48	34.59%	18/133	0.10 ± 0.30	13.53%
	48	38/133	0.00 ± 0.50	28.57%	14/133	0.11 ± 0.31	10.53%
	**P-value[2]**	0.727[1]	-	-	0.825[1]	-	-
**P-value[3]**	18	0.743[1]	-	-	0.428[1]	-	-
	28	0.791[1]	-	-	0.986[1]	-	-
	38	0.733[1]	-	-	0.431[1]	-	-
	48	0.062[1]	-	-	0.280[1]	-	-

[1]Chi-square test.

[2]Differences per tooth type within sex groups

[3]Males vs Females

Supplementary Table S3. Distribution of total number of third molar agenesis in males and females with and without agenesis of teeth other than third molars.

		**Without other teeth agenesis**		**With other teeth agenesis**		
**Female**	Number of missing third molars	Frequency	Percentage	Frequency	Percentage	**P-value*[4]**
	0	133/170	78.24%	81/170	47.65%	< 0.001[1]
	1	11/170	6.5%	14/170	8.2%	0.678[3]
	2	13/170	7.6%	24/170	14.1%	0.055[1]
	3	5/170	2.9%	18/170	10.6%	0.008[3]
	4	8/170	4.7%	32/170	18.9%	< 0.001[1]
		**Without other teeth agenesis**		**With other teeth agenesis**		
**Male**	Number of missing third molars	Frequency	Percentage	Frequency	Percentage	**P-value*[4]**
	0	108/133	81.20%	68/133	51.13%	< 0.001[1]
	1	9/133	6.8%	17/133	12.8%	0.098[1]
	2	6/133	4.5%	16/133	12%	0.026[1]
	3	1/133	0.8%	5/133	3.8%	0.098[3]
	4	9/133	6.8%	27/133	20.3%	0.001[1]
**P-value*[5]**	0	0.525[1]		0.547[1]		-
	1	0.917[1]		0.194[1]		-
	2	0.263[1]		0.594[1]		-
	3	0.235[3]		0.025[1]		-
	4	0.461[3]		0.747[1]		-

*Adjusted level of significance: $p < 0.01$

[1]Chi-square test, [2]Mann-Whitney test, [3]Fisher's exact test

[4]With versus without

[5]Males vs Females

Supplementary Table S4. Number of missing teeth per tooth type in males and females with agenesis of teeth other than third molars, considering all teeth of the dentition.

Tooth type	Females				Males				P-value*[4]
	Right	Left	P-value*	Total	Right	Left	P-value*	Total	
Lower 2nd PM	64	67	0.738[1]	131/340	53	50	0.802[1]	103/266	0.961[1]
Lower 3rd Molar	66	62	0.654[1]	128/340	38	46	0.291[1]	84/266	0.120[1]
Upper 3rd Molar	58	60	0.819[1]	118/340	43	45	0.794[1]	88/266	0.675[1]
Upper lateral incisor	44	45	0.902[1]	89/340	41	38	0.687[1]	79/266	0.336[1]
Upper 2nd PM	37	27	0.165[1]	64/340	23	25	0.749[1]	48/266	0.806[1]
Lower central incisors	19	19	1.000[1]	38/340	10	11	1.000[3]	21/266	0.176[1]
Lower 2nd Molar	11	9	0.818[3]	20/340	4	5	1.000[3]	9/266	0.152[1]
Upper 1st PM	10	10	1.000[3]	20/340	10	11	1.000[3]	21/266	0.327[1]
Lower 1st PM	9	9	1.000[3]	18/340	6	6	1.000[3]	12/266	0.659[1]
Lower lateral incisors	9	9	1.000[3]	18/340	6	9	0.596[3]	15/266	0.852[1]
Upper 2nd Molar	7	7	1.000[3]	14/340	6	7	1.000[3]	13/266	0.648[1]
Upper canines	5	6	1.000[3]	11/340	9	7	0.797[3]	13/266	0.300[1]
Lower 1st Molar	6	4	1.000[3]	10/340	3	3	1.000[3]	6/266	0.799[3]
Lower canines	2	2	1.000[3]	4/340	3	2	1.000[3]	5/266	0.515[3]
Upper 1st Molar	2	1	1.000[3]	3/340	3	3	1.000[3]	6/266	0.190[3]
Upper central incisors	1	1	1.000[3]	2/340	2	1	1.000[3]	3/266	0.658[3]

*Adjusted level of significance: $p < 0.003$

[1]Chi-square test, [2]Mann-Whitney test, [3]Fisher's exact test

[4]Sexual differences in the total number of missing teeth per tooth type
